# Cytokine gene expression can predict infectious complications following severe trauma

**DOI:** 10.1186/cc11965

**Published:** 2013-03-19

**Authors:** HD Torrance, K Brohi, CJ Hinds, MJ O'Dwyer

**Affiliations:** 1 Bart's Health NHS Trust, London, UK

## Introduction

Identifying a group of patients at high risk of developing infectious complications is the first step in the introduction of effective pre-emptive therapies in specific patient groups. Quantifying cytokine gene expression also furthers our understanding of trauma-induced immunosuppression. Our group has already demonstrated that a predictive immunological signature derived from mRNA expression in elective thoracic surgical patients accurately predicts pneumonia risk [1].

## Methods

In total, 121 ventilated polytrauma patients were recruited. mRNA was extracted from PaxGene tubes collected within 2 hours of the initial insult, at 24 and 72 hours. T-helper cell subtype specific cytokines and transcription factors mRNA was quantified using qPCR. Ten healthy controls served as a comparator.

## Results

**Figure 1:**
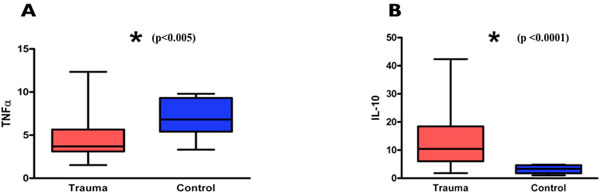
**Cytokine mRNA levels in trauma (time 0) and control groups**.

The Median Injury Severity Score (ISS) was 29. Time 0 bloods demonstrated a reduction in TNFα^†^, IL-12^§^, IL-23^‡^, RORγT* and T bet^§^, and an increase in IL-10* and IL-4^† ^mRNA levels in comparison with the control group (*P <0.0001, ^†^*P <*0.001 to 0.0001, ^‡^*P <*0.01 to 0.001, ^§^*P <*0.05 to 0.01). There was a positive correlation between ISS and IL-10^‡ ^whilst both IL-23^§ ^and RORγT^‡ ^were negatively correlated at time 0. TNFα^†^, IL-10* and IL-27^‡ ^increased and IFNγ^†^, IL-12*, IL-17A^§^, RORγT* and T bet* mRNA levels decreased over the initial 24 hours. Subsequent bacteraemia (18/121 patients) was associated with a lower TNFα/IL-10 ratio^‡ ^at baseline. Similarly, higher IL-10^‡ ^and lower T bet^‡ ^mRNA at 24 hours also predicted later bacteraemic episodes. Development of pneumonia followed a similar pattern. A multivariate logistical regression model proved highly accurate in predicting infectious complications from mRNA analysis of early blood samples. See Figure 1.

## Conclusion

Cytokine gene expression patterns indicate an immediate and sustained impairment in Th1, Th17 and innate immunity with concurrent upregulation of the Th2 response following major trauma. The magnitude of this response predicts subsequent infectious complications.
